# microRNA-497 prevents pancreatic cancer stem cell gemcitabine resistance, migration, and invasion by directly targeting nuclear factor kappa B 1

**DOI:** 10.18632/aging.204193

**Published:** 2022-07-25

**Authors:** Qiangfeng Yu, Zhe Xiu, Yizeng Jian, Jianyin Zhou, Xiaopeng Chen, Xiang Chen, Chunxiang Chen, Hongbao Chen, Sijia Yang, Libo Yin, Wenlong Zeng

**Affiliations:** 1 The Second Department of General Surgery, Zhuhai People’s Hospital, Zhuhai 51900, Guangdong, China; 2 Department of Hepatobiliary Surgery, The Second Hospital of Longyan, Longyan 364000, Fujian, China; 3 Department of Hepatobiliary and Pancreatic Surgery, Zhongshan Hospital, Xiamen University, Xiamen 361000, Fujian, China; 4 The Third Department of Surgery, The Second Hospital of Longyan, Longyan 364000, Fujian, China; 5 Department of Science and Education, The Second Hospital of Longyan, Longyan 364000, Fujian, China; 6 Department of Pathology, The Second Hospital of Longyan, Longyan 364000, Fujian, China; 7 The First People’s Hospital of Wenling, The Affiliated Wenling Hospital of Wenzhou Medical University, Wenzhou 317500, Zhejiang, China

**Keywords:** microRNA-497, nuclear factor kappa B 1, pancreatic cancer, cancer stem cells, gemcitabine

## Abstract

Objectives: Cancer stem cells (CSCs) comprise a small population of cells in cancerous tumors and play a critical role in tumor resistance to chemotherapy. miRNAs have been reported to enhance the sensitivity of pancreatic cancer to chemotherapy. However, the underlying molecular mechanism requires better understanding.

Methods: Cell viability and proliferation were examined with CCK8 assays. Quantitative real-time polymerase chain reaction was executed to assess mRNA expression. StarBase database was used to select the target genes of miRNA, which were further affirmed by dual luciferase assay. Transwell assay was used to analyze cell invasion and migration.

Results: We proved that miR-497 could be obviously downregulated in pancreatic cancer tissues and CSCs from Aspc-1 and Bxpc-3 cells. In addition, inhibition of miR-497 evidently accelerated pancreatic CSC gemcitabine resistance, migration and invasion. Moreover, we revealed that nuclear factor kappa B 1 (NFκB1) was prominently upregulated in pancreatic cancer tissues and pancreatic CSCs, and NFκB1 was also identified as a direct target of miR-497. Furthermore, we demonstrated that overexpression of NFκB1 could also notably promote the viability, migration, and invasion of gemcitabine-treated pancreatic CSCs, but this effect could be partially abolished by miR-497 overexpression.

Conclusions: Those findings suggest that miR-497 overexpression could suppress gemcitabine resistance and the metastasis of pancreatic CSCs and non-CSCs by directly targeting NFκB1.

## INTRODUCTION

Pancreatic cancer is the fourth leading cause of cancer-related death in developed countries and is predicted to reach second place within several years [1, 2]. Most pancreatic tumors are unresectable upon diagnosis due to the presence of local advanced disease or distant metastases [3]. These cancers are characterized by high resistance to most conventional therapies, such as chemotherapy, radiotherapy, and molecular targeted therapy [4]. Systemic chemotherapy with gemcitabine is the standard first-line treatment for pancreatic cancer, but the clinical response rate is only 23.8%, the 5-year patient survival rate is 2%, and the 1-year survival rate ranges between 17 and 23% [5–7]. Improving a tumor’s sensitivity to gemcitabine is considered a promising strategy for increasing the therapeutic efficacy of that anticancer drug. Thus, there remains an urgent need to understand the mechanisms that underlie the innate or acquired resistance to gemcitabine displayed by pancreatic tumors.

Various types of cells exist within a malignant tumor. Cancer stem cells (CSCs) comprise a small population of cells that are capable of self-renewal and differentiation into all cell types that comprise a particular tumor [8]. Increasing evidence suggests that CSCs play a critical role not only in tumor development, metastasis, and cancer relapse but also in a tumor’s resistance to methods of treatment, such as chemotherapy and radiotherapy [9–11]. A recent study showed that CSC enrichment in Panc-1 cells enhanced cell migration and resistance to gemcitabine [12]. Another study also revealed gemcitabine resistance in pancreatic CSCs [13]. Furthermore, treatments targeting CSCs not only inhibited tumor growth but also increased the sensitivity of pancreatic CSCs and tumors to gemcitabine [14]. However, the mechanism by which CSCs develop resistance to gemcitabine remains largely unknown.

MicroRNAs (miRNAs) comprise a family of small single-stranded, evolutionarily conserved noncoding RNAs [15, 16]. MiRNAs bind to their target RNA molecules to regulate gene expression at the translational level and also regulate various physiological and pathological processes, including cell proliferation, apoptosis, invasion, migration, differentiation, metastasis, and angiogenesis [16, 17]. MicroRNA-497 (miR-497), a member of the miR-15 family, is detectable in almost all human organs and tissues [18]. Decreased levels of miR-497 expression have been reported in a variety of cancers, including pancreatic, breast, and lung cancers [19–21]. Furthermore, the downregulation of miRNA levels is considered associated with tumor malignancy and a poor prognosis [22]. Therefore, miR-497 expression might exert a direct anticancer effect. In addition, miR-497 was shown to increase the sensitivity of some tumors to chemotherapy; for example, it increased the sensitivity of colorectal cancer cells to 5-fluorouracil and increased the sensitivity of pancreatic cancer cells to both gemcitabine and erlotinib [23, 24]. Recent studies have also confirmed that miRNAs play a key role in the chemosensitization of pancreatic CSCs. For instance, miR-137 could decrease the stemness of pancreatic cancer cells [25]; miR-30b could block the epithelial-mesenchymal transition of pancreatic CSCs [26]; and miR-205 could induce gemcitabine resistance in pancreatic CSCs [27]. Therefore, we hypothesized that miR-497 might play a role in regulating the sensitivity of pancreatic CSCs to various types of chemotherapy.

Through bioinformatics screening and identification, we preliminarily confirmed that nuclear factor kappa B 1 (NFκB1, p105/p50) is a potential target gene of miR-497. NFκB1 is a subunit of NF-κB, whose aberrant activation is considered associated with cancer pathogenesis [28, 29]. NFκB1 is initially formed as a precursor molecule (p105) and then proteolytically cleaved to p50. It has been reported that NFκB1 plays multiple roles in the development and progression of different cancers [30]. For example, NFκB1-/- mice develop fewer colorectal tumors, indicating that NFκB1 acts as a tumor promoter [31]. In contrast, NFκB1 also acts as a tumor suppressor in some tumors, such as hepatocellular carcinoma and gastric cancer. The role of p50 in carcinogenesis remains controversial [32]. Jung et al. [24] reported that increased p50 signaling was associated with cancer pathogenesis [33]. Another study conducted in a mouse model of pancreatic cancer suggested a potential role for p50 in pancreatic cancer prevention [34]. However, the effects of NFκB1 on pancreatic CSCs and the related mechanisms have not been reported.

In the present study, we further investigated the impacts of miR-497 inhibition on the gemcitabine resistance, migration, and invasion of pancreatic CSCs. In addition, we verified a potential mechanism by which miR-497 affects the gemcitabine resistance and metastasis of pancreatic CSCs. NFκB1 was first identified as a direct target of miR-497, which could reverse the effect of miR-497 on pancreatic CSCs. Therefore, our findings provide novel insight into the mechanism underlying the high resistance of pancreatic cancer to gemcitabine and the metastasis of pancreatic cancer, which might suggest miR-497 as a novel adjuvant therapy for pancreatic cancer.

## MATERIALS AND METHODS

### Tissue

From October 2017 to May 2019, 30 cases of pancreatic cancer in patients aged 32-70 years (average 56 years) were treated. All cases followed the international diagnosis of pancreatic cancer and were confirmed by pathology. In addition, fresh tumor tissue samples were collected from all these patients, and matched adjacent tissue samples were used as controls based on a previous study [35]. The inclusion criteria included patients undergoing radical operation, histopathological confirmation of pancreatic ductal adenocarcinoma, no other malignant tumors, no radiation or chemotherapy, and complete clinical data. The exclusion criteria included patients who did not undergo surgery, patients undergoing palliative surgery, patients with no pathological diagnosis or unclear diagnosis, and concomitant malignancies; the clinical features were not perfectly distributed. There were 6 males and 9 females; 3 patients with tumor size ≥ 3 cm, 12 patients with tumor size <3 cm; 8 patients with age ≥58, 7 patients with age<58; 13 patients with TNM (I+II), 2 patients with TNM (III+IV) (Table 1). The study was approved by the Ethics Committee of Zhuhai People’s Hospital, and written consent was obtained from all selected patients.

**Table 1:** Association of miR-497 expression and clinicopathologic characteristics in pancreatic cancer patients.

**Clinical features**		**MiR-497 expression**		***p* value**
		**High expression**	**Low expression**	
Gender				>0.999
	Male	6	6	
	Female	9	9	
Tumor size (cm)				
	≥3	3	12	**0.003
	<3	12	3	
Age				>0.999
	≥58	8	7	
	<58	7	8	
TNM				***0.001
	I+II	13	3	
	III+IV	2	12	

### Cell culture and transfection

The human pancreatic cell lines BxPC-3 and AsPC-1 were obtained from the Cell Bank in the Type Culture Collection Center of the Chinese Academy of Sciences (Shanghai, China). Both cell lines were maintained in RPMI-1640 medium (Gibco, Pittsburg, PA, USA) containing 10% fetal bovine serum (FBS; Gibco) and 1% penicillin/streptomycin (Life Technologies, Carlsbad, CA, USA) at 37° C in a humidified atmosphere with 5% CO_2_. MiRNA mimics, a miRNA inhibitor, a pCDNA3.1 vector containing NFκB1 cDNA, and the corresponding negative controls (NCs) were obtained from Sangon Biotech (Shanghai, China). A mimic of miR-497 was synthesized using the following primers: Forward, 5′- CAGCAGCACACUGUGGUUUGU-3’. To downregulate miR-497 in BxPC-3 and AsPC-1 cells, an inhibitor of miR-497 was synthesized using the following single-stranded RNA: 5′-ACAAACCACAGUGUGCUGCUG-3′. A control mimic of a random miR-497 sequence (forward, 5’-UCGAGUCGCUCACUGUUACCC-3’) and a control inhibitor of a random miR-497 sequence (5’-CUAGAUGGCACACACGAGGCU-3’) were used as NCs. BxPC-3 and AsPC-1 cells were transfected by using Lipofectamine 2000 (Invitrogen, Carlsbad, CA, USA) according to the manufacturer’s instructions. Sulforaphane was obtained from Sigma (Sigma–Aldrich, Beijing, China, 4478-93-7).

### Preparation of CSCs

CSCs were prepared as previously described [36]. In brief, BxPC-3 and AsPC-1 cells were collected and adjusted to 1×10^10^ cells/ml. Then, 20 μl anti-CD24-PE (1:40, Abcam), anti-CD44-APC (1:40, Abcam) and anti-ESA-FITC (1:40, Abcam) were added to the cells for 20 min. The control group was not given antibodies. After centrifugation and suspension, the positive cells were sorted by flow cytometry. Negative cells were used as non-CSCs. Then, the cells were cultured in flasks containing serum-free Dulbecco’s modified Eagle medium (DMEM)/F12 supplemented with 10 ng/mL fibroblast growth factor 4 (FGF-4), 20 ng/mL epidermal growth factor (EGF), 0.2 U/mL insulin (Sigma–Aldrich, Beijing, China), 1% N_2_ supplement, and 2% B27 supplement. After 10-14 days, clusters of spherical cells formed that were subsequently enzymatically dissociated into single cells. The dissociated single cells and nonspherical cells (both densities = 1000 cells/mL) were separately maintained in low attachment culture plates containing serum-free medium (SFM) and subcultured every 10-14 days. The special clusters of cells (CD24^+^CD44^+^ESA^+^) cultured under these conditions were designated CSCs. Negative cells (CD24^-^CD44^-^ESA^-^) were used as non-CSCs. CSCs and non-CSCs from AsPC-1 and BxPC-3 cells were treated with 0, 2, 4, 6, 8, 10, and 12 μM gemcitabine; CSCs and non-CSCs from AsPC-1 and BxPC-3 cells were treated with 0, 0.1, 0.2, 0.4, 0.8, 1.6, and 3.2 μM gemcitabine.

### Cell counting kit-8 (CCK-8) assay

Aiquots of suspended 1 × 10^5^ cells (1 μL) were seeded into the wells of 96-well plates. After overnight culture, the cells were exposed to gemcitabine at the designed concentrations, and cell viability was measured 12 h or 24 h later. CCK-8 reagent 10 μL; (Beyotime, Haimen, China) was added to each well, and after one hour of incubation, the optical density of each well at 450 nm was determined with a spectrophotometer (Bio–Rad, Hercules, CA, USA). The experiment was repeated three times.

### RNA extraction and real-time quantitative RT-PCR

Total RNA was extracted from pancreatic cancer cells, CSCs, and non-CSCs using TRIzol Reagent (Invitrogen) and then quantified by spectrophotometry. The RNA was then reverse transcribed into cDNA by using PrimeScript RT Reagents (TaKaRa, Dalian, China) according to the manufacturer’s protocol. RT–PCR was performed on triplicate samples by using a GoTaq® qPCR Master Mix kit (Promega, Madison, WI, USA) on an ABI 7500 Real-time PCR System (Applied Biosystems, Foster City, CA, USA). The reaction protocol was as follows: initial denaturation at 94° C for 2 min, followed by 40 cycles of denaturation at 94° C for 30 s, annealing at 60° C for 20 s, and elongation at 68° C for 20 s. The relative levels of gene expression were determined by the 2^-ΔΔCT^ method [37]. The GAPDH mRNA and U6 small nuclear RNA were used as internal controls, respectively. The primer sequences were the following: miR-497 R: CTC AAC TGG TGT CGT GGA; miR-497-F: ACA CTC CAG CTG GGC AGC AGC ACA CTG TGG T; U6-F: CTC GCT TCG GCA GC ACA; U6-R: AAC GCT TCA CGA ATT TGC GT; NFkB1-F: AAC AGA GAG GAT TTC GTT TCC G; NFkB1-R: TTT GAC CTG AGG GTA AGA CTT CT; GAPDH-F: TGT TCG TCA TGG GTG TGA AC; GAPDH-R: ATG GCA TGG ACT GTG GTC AT; MMP2-F: TAC AGG ATC ATT GGC TAC ACA CC; MMP2-R: GGT CAC ATC GCT CCA GAC T; Ki67-F: ACG CCT GGT TAC TAT CAA AAG G; Ki67-R: CAG ACC CAT TTA CTT GTG TTG GA; CD147-F: GAA GTC GTC AGA ACA CAT CAA CG; CD147-R: TTC CGG CGC TTC TCG TAG A;.

### Cell migration and invasion assays

Cell migration and invasion assays were conducted using 24-well Transwell plates with 8-μm pore size chamber inserts (Millipore, Billerica, MA, USA). The invasion assays were performed using chamber inserts precoated with 0.1% collagen (w/v), while migration assays were performed using noncoated chamber inserts. In brief, cells (2 x 10^4^) that had been treated with gemcitabine were added to the chamber inserts with 0.2 mL of serum-free RPMI-1640 medium containing 2% FBS, and medium containing 15% FBS was added to the lower chamber. After 24 h of incubation, cells on the top surface of the chamber inserts were carefully removed, while cells on the bottom surface were treated with 4% paraformaldehyde for 15 min and then stained with 0.1% crystal violet for 20 min. The stained cells were viewed under a light microscope (Olympus, Tokyo, Japan).

### Luciferase reporter assay

By referring to previous research, the wild-type (WT) or mutant (MUT) 3′-UTR of NFκB1 mRNA with the predicted binding site for miR-497 was amplified using PCR and then cloned into a psiCHECK-2 vector (Promega). The cloned vectors were validated by sequencing. Aspc-1 and BXPC-3 cells were seeded into 24-well plates. After being cultured overnight, the cells were cotransfected with plasmids, miR-497 mimics or NC using Lipofectamine 2000. At 24 h post-transfection, luciferase activity was measured and normalized to that of the internal control.

### Animal experiments

Male BALB/c nude mice (4 weeks old) were randomly divided. CSCs and non-CSCs from AsPC-1 were subcutaneously inoculated into the right flank of each mouse (200 μL, 5*10^6^ cells/mL). Tumor size was monitored weekly. The tumor volumes were calculated according to the formula: volume=0.5* width^2^*length. Animal procedures in this study were supervised by The Second Hospital of Longyan.

### Statistical analysis

All data were analyzed using SPSS for Windows, Version 13.0 software (SPSS Inc., Chicago, IL, USA), and the results are expressed as the mean value ± SD of data obtained from at least three independent experiments. The statistical significance of differences between groups was assessed by Student’s *t-test*, and multiple comparisons were made using one-way ANOVA followed by Tukey’s test. P<0.05 was considered to indicate a statistically significant difference.

## RESULTS

### Expression of miR-497 in pancreatic CSCs

**Figure 1:**
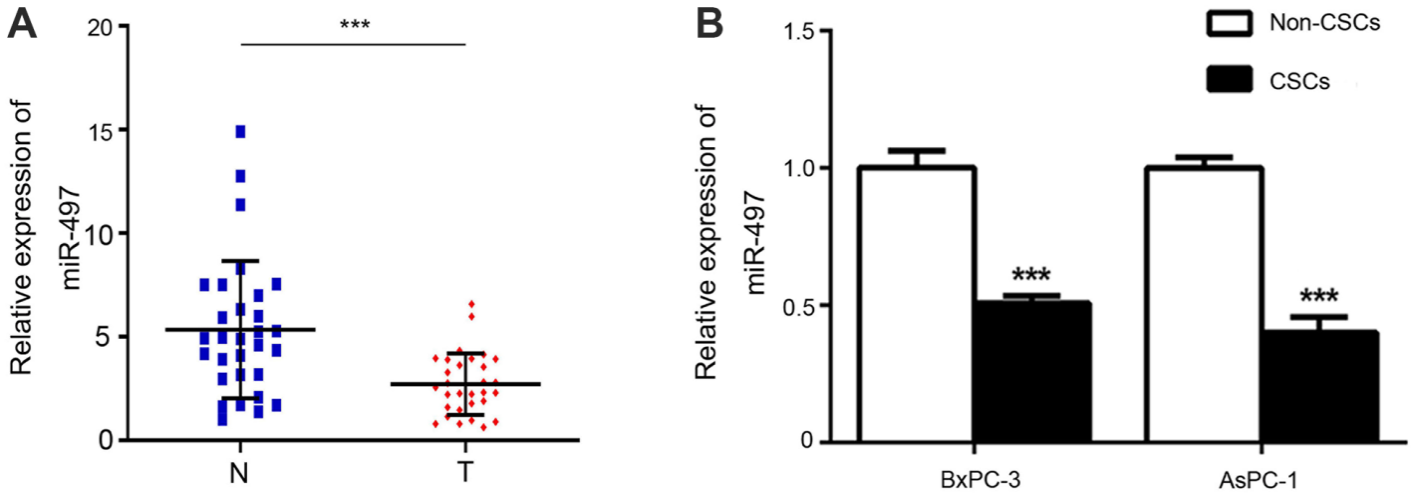
**Expression of miR-497.** The levels of miR-497 in tissues (**A**) and cells (**B**) were determined by RT–PCR. N, normal; T, tumor; CSCs, cancer stem cells. ***p < 0.001, as compared with non-CSCs.

To investigate the role of miR-497 in pancreatic CSCs, we first verified the expression changes in miR-497 in pancreatic cancer tissues and CSCs and non-CSCs from Aspc-1 and Bxpc-3 cells. As shown in Figure 1A, miR-497 expression was significantly decreased in pancreatic cancer tissues compared with normal tissues. In addition, we discovered that high miR-497 expression was related to small tumor size (P=0.003) and low TNM stage (P<0.001) (Table 1). As for Aspc-1 and Bxpc-3 cells, CSCs and non-CSCs were prepared, and subsequent analysis showed that miR-497 was expressed at lower levels in the CSCs (Figure 1B). Taken together, these findings indicated that miR-497 is aberrantly expressed in pancreatic cancer cells and tissues and might be associated with certain characteristics of CSCs.

### Gemcitabine resistance analysis of CSCs and non-CSCs from AsPC-1 and BxPC-3 cells

**Figure 2:**
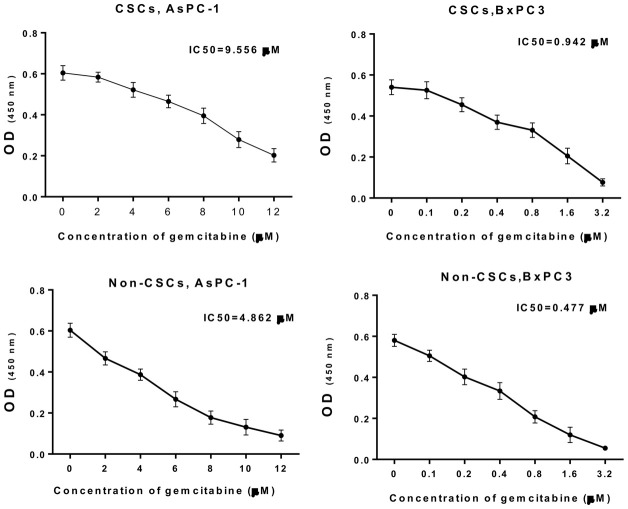
**Gemcitabine resistance analysis of CSCs and non-CSCs from AsPC-1 and BxPC-3 cells.** CSCs and non-CSCs from AsPC-1 and BxPC-3 cells were processed with different concentrations of gemcitabine for 24 h. Cell growth was determined with CCK-8 assay, and the IC50 values were calculated.

In addition, CSCs and non-CSCs were isolated from AsPC-1 and BxPC-3 cells, which were also treated with different concentrations of gemcitabine, and cultured. The CCK-8 data (Figure 2) indicated that with increasing drug concentration, cell viability gradually decreased. The half-maximal inhibitory concentration (IC50) for CSCs from AsPC-1 cells was 9.556 μM, the IC50 for CSCs from BxPC-3 cells was 0.942 μM, the IC50 for non-CSCs from AsPC-1 cells was 4.862 μM, and the IC50 for non-CSCs from BxPC-3 cells was 0.477 μM. Based on the concentration screening, non-CSCs from AsPC-1 cells were treated with 2 μM gemcitabine; CSCs from AsPC-1 cells were treated with 4 μM gemcitabine; no-CSCs from BxPC-3 cells were treated with 0.2 μM gemcitabine; CSCs from BxPC-3 cells were treated with 0.4 μM gemcitabine in the follow-up experiments.

### Inhibition of miR-497 contributed to gemcitabine resistance

**Figure 3:**
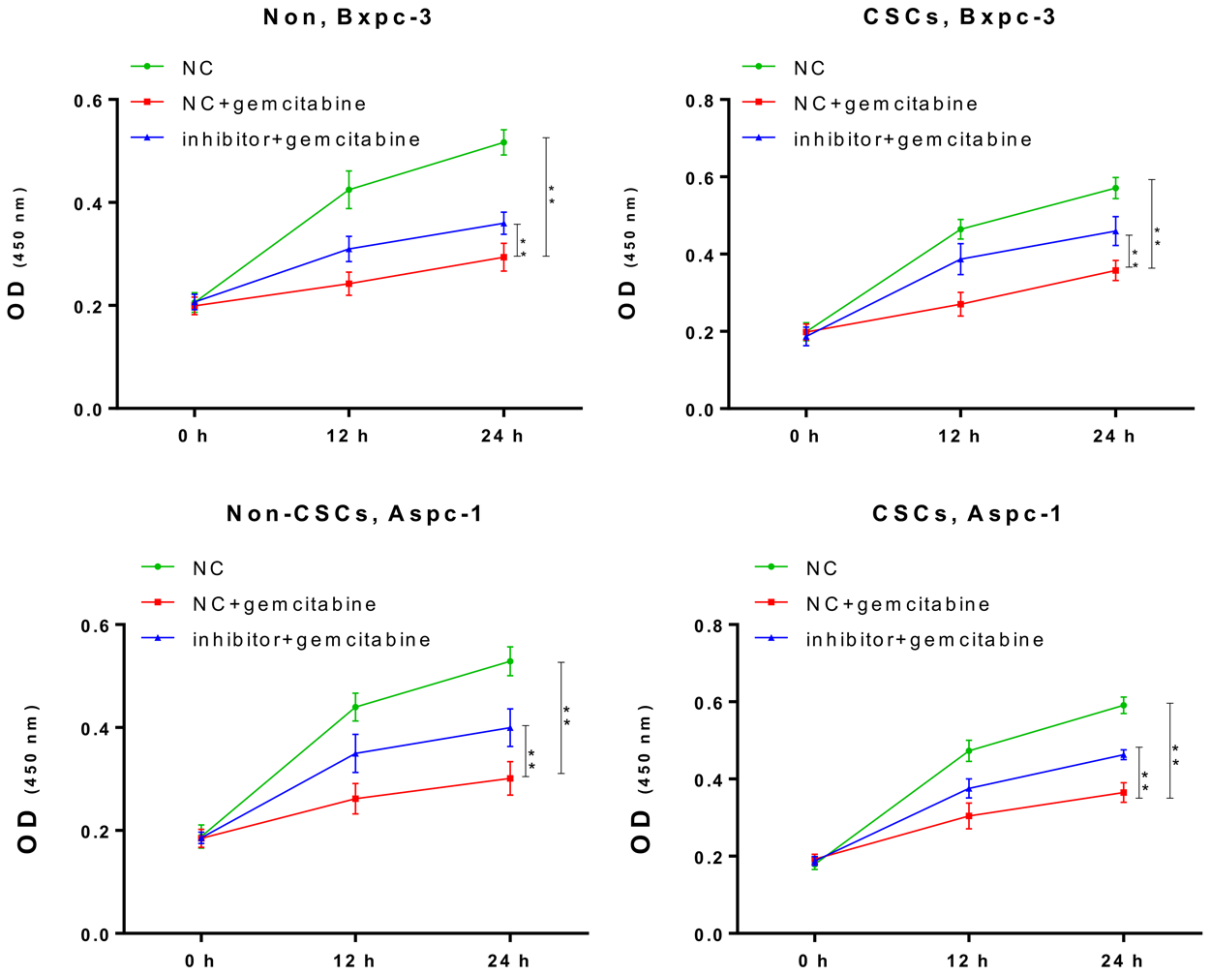
**The miR-497 inhibitor significantly reduced the cytotoxicity of gemcitabine.** The CSCs or non-CSCs of AsPC-1 and BxPC-3 cells transfected with the NC or miR-497 inhibitor were treated with gemcitabine for 0, 12, or 24 h, after which cell viability was determined by CCK-8 assay NC, negative control; CSCs, cancer stem cells; *p < 0.05, **p < 0.01 compared with NC + gemcitabine.

Next, we further confirmed the impact of miR-497 inhibition on the gemcitabine resistance of CSCs and non-CSCs from Aspc-1 and Bxpc-3 cells. After transfection with the miR-497 inhibitor, a significant increase in the viability of both CSCs and non-CSCs was observed (Figure 3). This suggested that miR-497 inhibition leads to gemcitabine resistance that might be associated with the gemcitabine resistance property of CSCs in populations of ASPC-1 and Bxpc-3 cells.

### miR-497 inhibition notably induced the migration and invasion of pancreatic CSCs and non-CSCs

**Figure 4:**
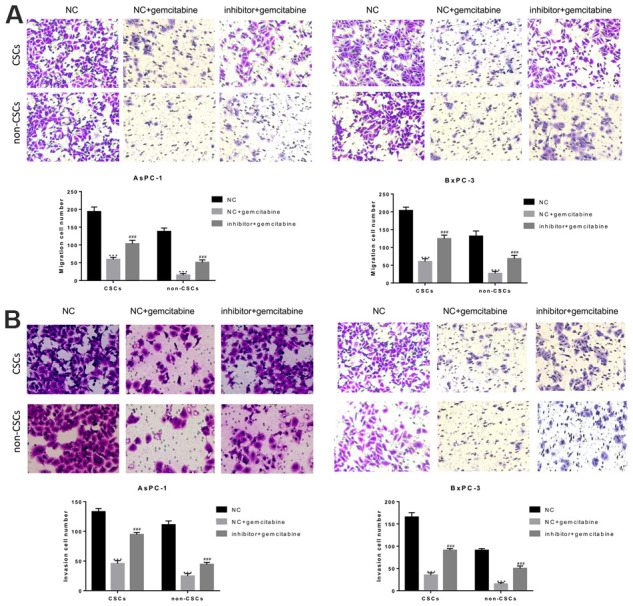
**The miR-497 inhibitor significantly inhibited gemcitabine-induced decreases in CSC and non-CSC migration and invasion.** CSCs and non-CSCs isolated from populations of AsPC-1 and BxPC-3 cells were transfected with NC or miR-497 inhibitor and then exposed to gemcitabine for 24 h. The migration (**A**) and invasion (**B**) capabilities of the cells were examined. ***P< 0.001, compared to the NC group; ###P< 0.001, compared to the NC+gemcitabine group. Data are shown as mean ± SEM. All experiments were repeated at least three independent experiments.

To further examine the roles played by miR-497 in the migration and invasion of CSCs and non-CSCs from Aspc-1 and Bxpc-3 cells, the cells were transfected with a miR-497 inhibitor, and cell migration and invasion were examined. As shown in Figure 4A, the migration of both CSCs and non-CSCs was significantly reduced after gemcitabine treatment compared with that of cells that received NC treatment. This inhibitory effect was significantly suppressed in cells transfected with the miR-497 inhibitor. Likewise, a reduction in gemcitabine-induced inhibition of cell invasion was noted after miR-497 transfection (Figure 4B).

### miR-497 inhibitor increased the expression of the viability, migration, and invasion biomarker genes

**Figure 5:**
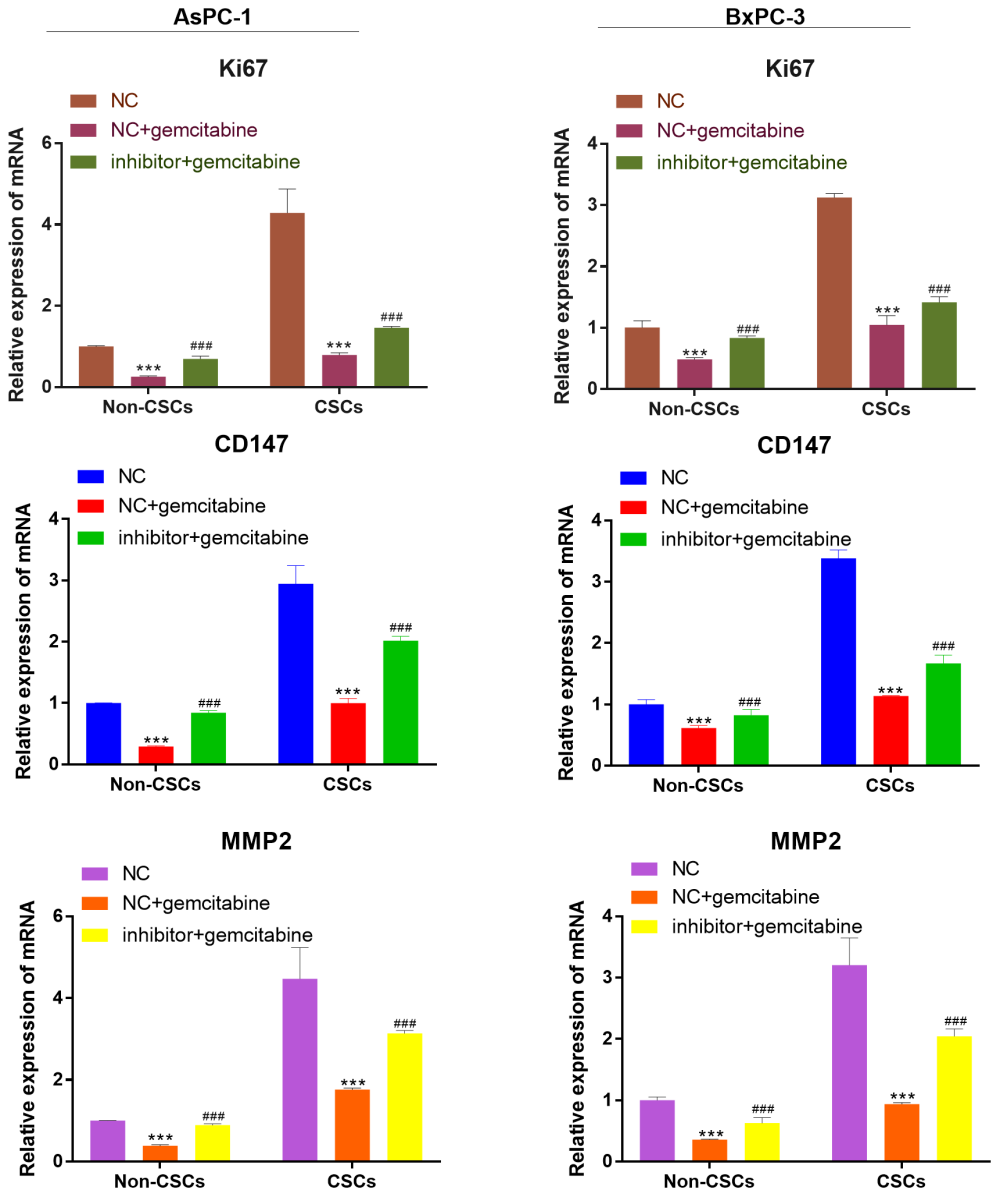
**miR-497 inhibitor increased the expression of the viability, migration, and invasion biomarker genes.** qRT-PCR assay analysis of Ki67, CD147, MMP-2 expression level in CSCs and non-CSCs isolated from populations of AsPC-1 and BxPC-3 cells. ***P< 0.001, compared to the NC group; ###P< 0.001, compared to the NC+gemcitabine group. Data are shown as mean ± SEM. All experiments were repeated at least three independent experiments.

As detected by RT-qPCR, the mRNA expression of viability, migration, and invasion biomarker genes (Ki67, CD147, MMP-2) were markedly reduced after gemcitabine treatment compared with that of cells that received NC treatment. However, this expression was reversed by miR-497 inhibitor (Figure 5).

### NFκB1 was identified as a direct target of miR-497

**Figure 6:**
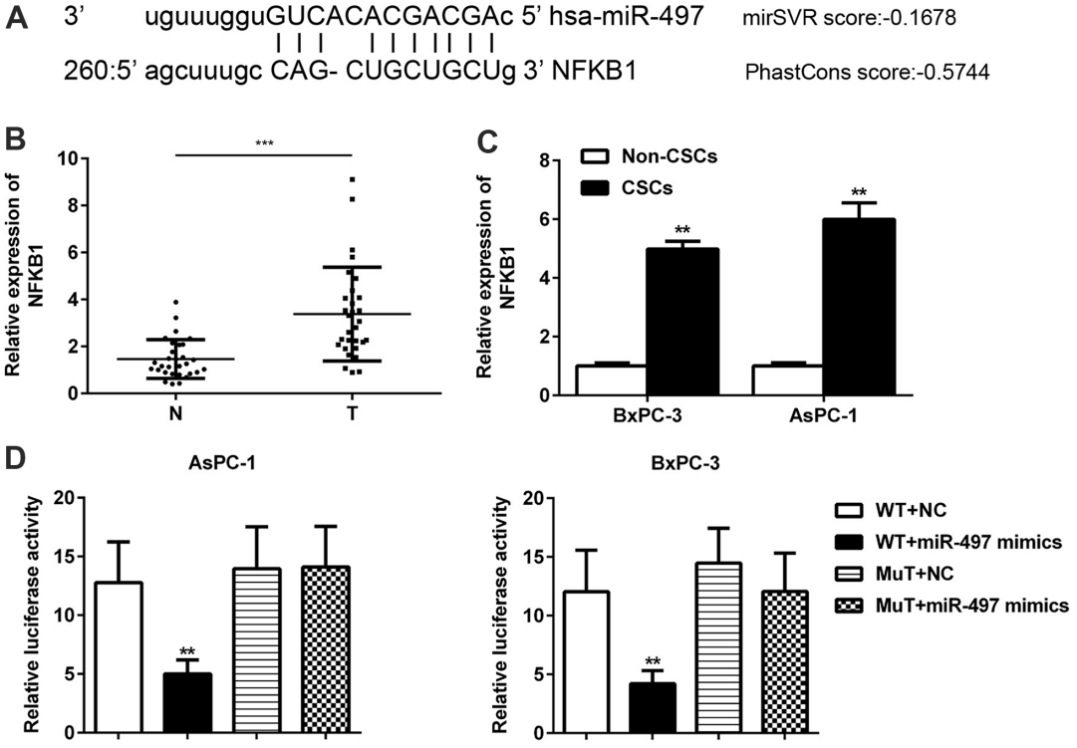
**NFκB1 was identified as a direct target of miR-497.** The binding sites for miR-497 and NFκB1 were predicted using miRanda software (**A**). The levels of NFκB1 expression were examined in pancreatic cancer (**B**) and in CSCs and non-CSCs from pancreatic cancer cell lines (**C**). The interaction between miR-497 and NFκB1 in AsPC-1 and BxPC-3 cells was examined by luciferase reporter assays (**D**). N, normal pancreatic tissue; T, pancreatic tumor; CSCs, cancer stem cells; Mut, mutant; WT, wild type.

In addition, we further searched and verified the potential target genes of miR-497 using the bioinformatics software package miRanda. The data showed that miR-497 contained binding sites for NFκB1 mRNA (Figure 6A). In contrast to miR-497 levels, we found significant increases in the levels of NFκB1 in CSCs when compared with normal tissues (Figure 6B). A comparison of CSCs and non-CSCs prepared from Aspc-1 and Bxpc-3 cells revealed significantly higher levels of NFκB1 in the CSCs (Figure 6C). These data suggested that NFκB1 might be negatively regulated by miR-497. A further assay showed that miR-497 mimics significantly decreased luciferase activity in the NFκB1 3’-UTR WT but had no effect on luciferase activity in the 3’-UTR MT (Figure 6D). These findings suggest that NFκB1 is a direct target of miR-497.

### miR-497 markedly decreased NFκB1-induced resistance to gemcitabine in pancreatic CSCs and non-CSCs

**Figure 7:**
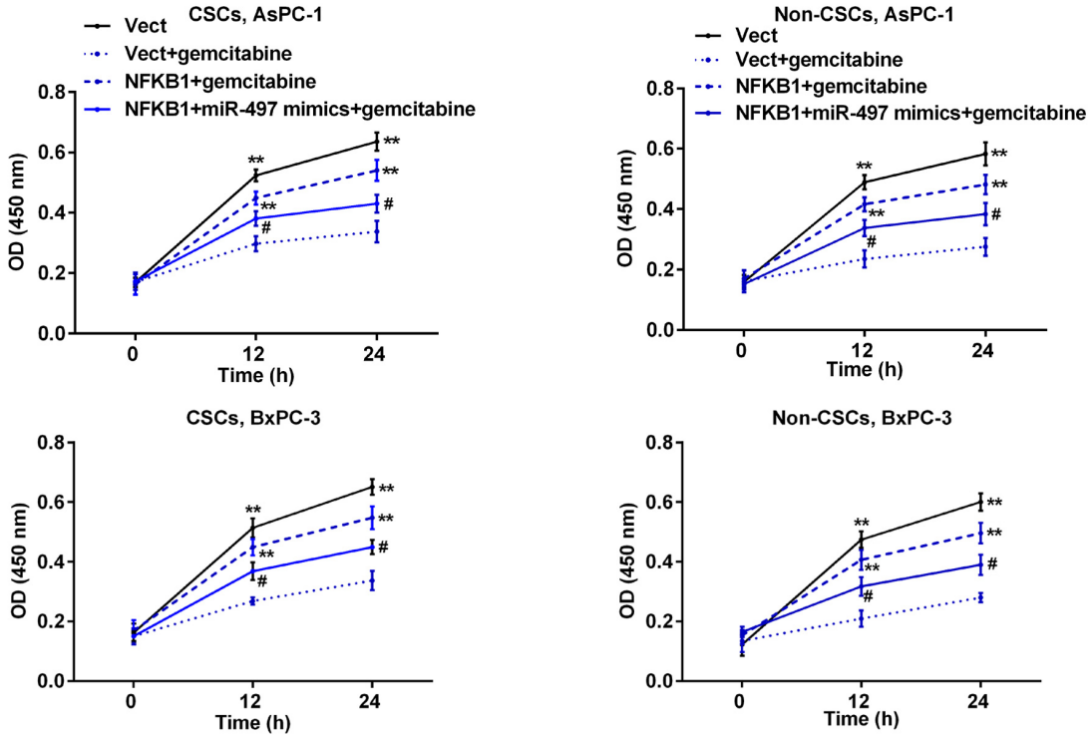
**MiR-497 mimics inhibited NFκB1-induced resistance to gemcitabine in pancreatic cancer cells.** CSCs and non-CSCs derived from populations of AsPC-1 or BxPC-3 cells were transfected with the NFκB1 overexpression vector, empty vector alone, or in combination with miR-497 mimics and then exposed to gemcitabine. After 24 h of treatment, cell viability was examined by the CCK-8 assay. **p < 0.01, compared to the Vect + gemcitabine group; #p < 0.05, compared to the NFκB1+gemcitabine group. Vect, empty vector; CSCs, cancer stem cells.

Subsequently, we further examined how NFκB1 can affect the sensitivity of pancreatic cells to gemcitabine. Pancreatic cells were transfected with the NFκB1 overexpression plasmid and then exposed to gemcitabine. As shown in Figure 7, transfection with the NFκB1 overexpression plasmid significantly increased the viability of CSCs and non-CSCs prepared from Aspc-1 and Bxpc-3 cells when compared to cells transfected with the empty vector, suggesting that NFκB1 helps to protect cells against the cytotoxic effects of gemcitabine. However, this protective effect was markedly attenuated by additional transfection with miR-497 mimics.

### miR-497 overexpression strongly suppressed migration and invasion mediated by NFκB1 overexpression in pancreatic CSCs and non-CSCs

**Figure 8:**
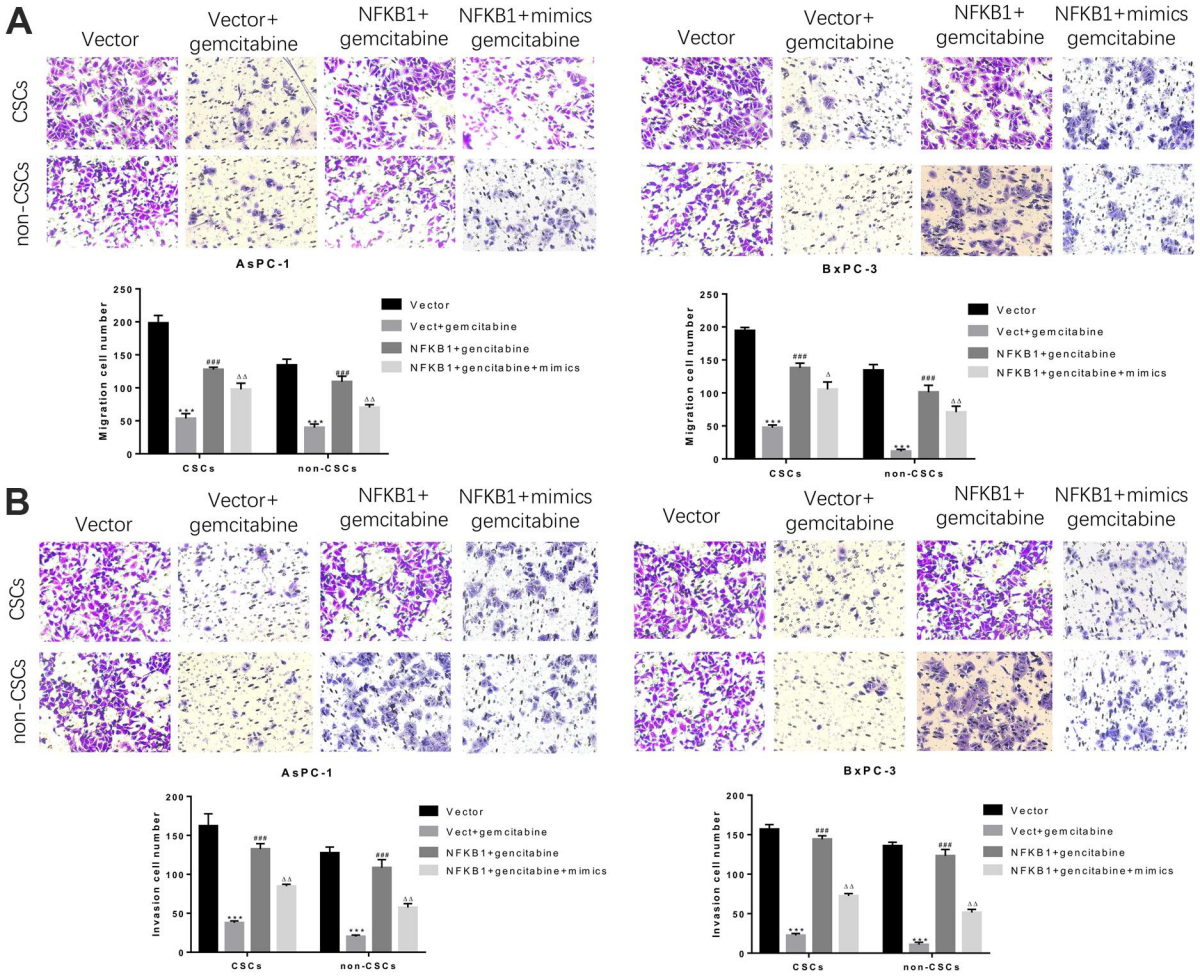
**MiR-497 mimics significantly inhibited gemcitabine-induced decreases in pancreatic cancer cell migration and invasion.** CSCs and non-CSCs derived from populations of AsPC-1 or BxPC-3 cells were transfected with an NFκB1 overexpression vector, empty vector alone, or in combination with miR-497 mimics and then exposed to gemcitabine. After 24 h of treatment, the migration (**A**) and invasion (**B**) capabilities of the cells were examined. ***P< 0.001, compared to the Vector group; ###P< 0.001, compared to the Vector+gemcitabine group. ∆∆P<0.01, Vector+gemcitabine group. Data are shown as mean ± SEM. All experiments were repeated at least three independent experiments.

Similarly, we further assessed whether NFκB1 could weaken the inhibitory effect of miR-497 on the migration and invasion of CSCs and non-CSCs from Aspc-1 and Bxpc-3 cells. The results confirmed that NFκB1 transfection significantly attenuated the gemcitabine-induced reductions in cell migration and invasion, and those effects were markedly suppressed by transfection with miR-497 mimics. All these findings indicated that overexpression of NFκB1 confers gemcitabine resistance, which could be attenuated by miR-497 mimics (Figure 8A, 8B).

### miR-497 inhibited the expression of the viability, migration, and invasion biomarker genes

**Figure 9:**
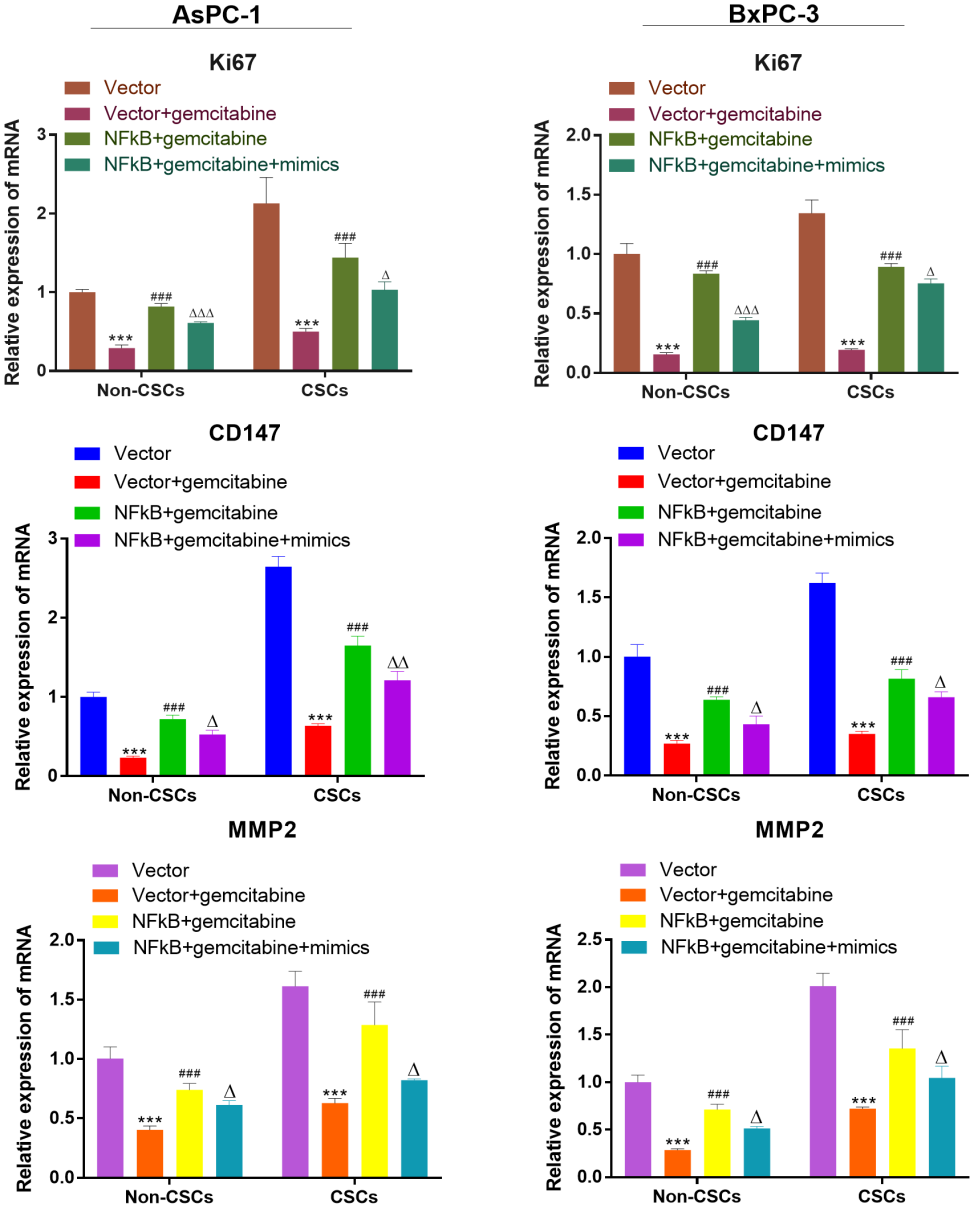
**miR-497 inhibited the expression of the viability, migration, and invasion biomarker genes.** qRT-PCR assay analysis of Ki67, CD147, MMP-2 expression level in CSCs and non-CSCs isolated from populations of AsPC-1 and BxPC-3 cells. ***P< 0.001, compared to the Vector group; ###P< 0.001, compared to the Vector+gemcitabine group. ∆∆P<0.01, Vector+gemcitabine group. Data are shown as mean ± SEM. All experiments were repeated at least three independent experiments.

We also investigate the expression of viability, migration, and invasion biomarker genes (Ki67, CD147, MMP-2) in CSCs and non-CSCs from Aspc-1 and Bxpc-3 cells. The mRNA expression of Ki67, CD147 and MMP-2 were decreased in NFκB1 transfection group. However, the mRNA expression of those genes increased by miR-497 administration (Figure 9).

### CSCs promotes tumor growth *in vivo* than in non-CSCs

**Figure 10:**
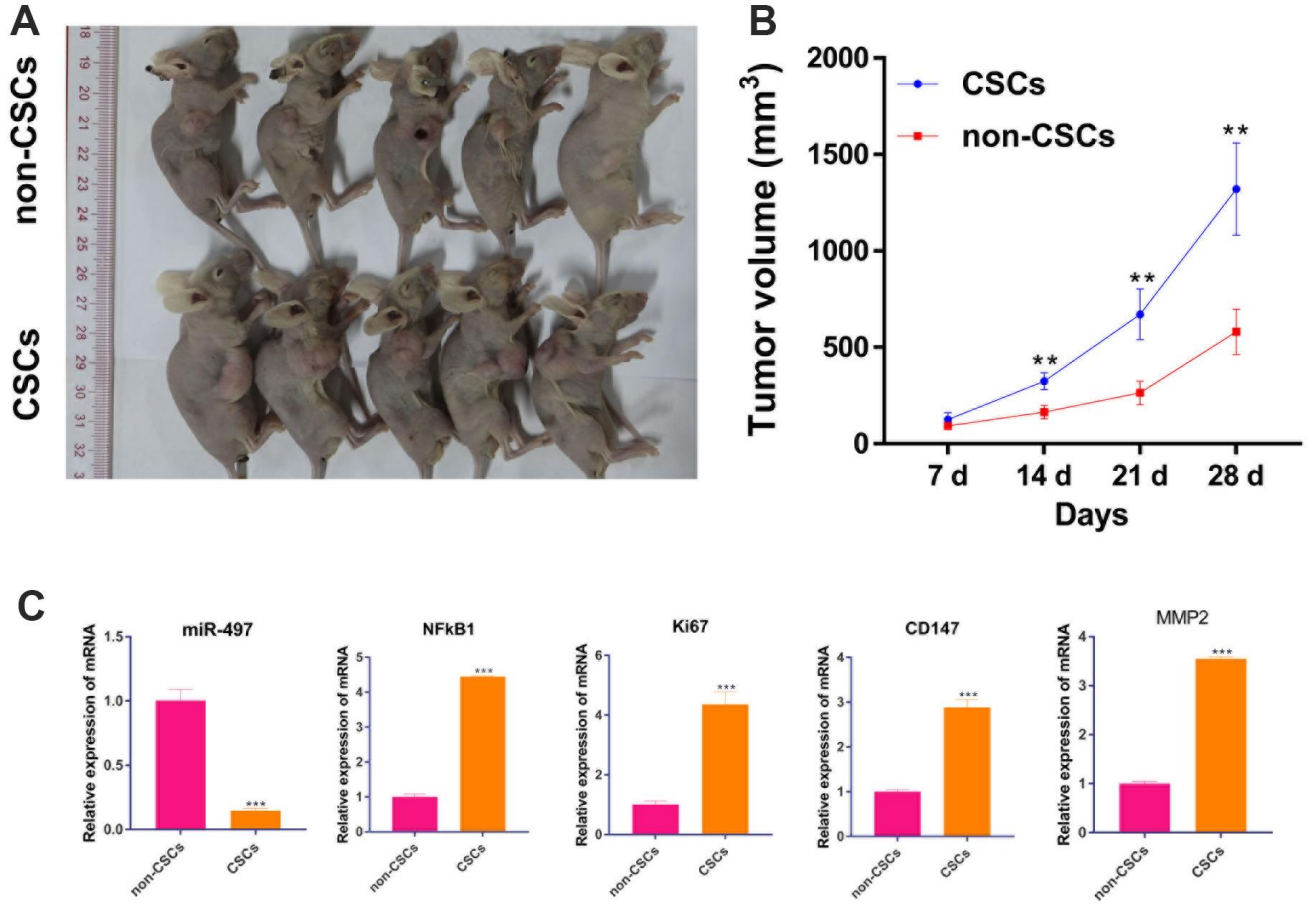
**CSCs promotes tumor growth *in vivo* than in non-CSCs.** A mouse model of Pancreatic cancer was established by subcutaneously inoculating CSCs and non-CSCs. (**A**) xenograft tumors of each group were shown; (**B**) Tumor volume was measured every week. (**C**) The mRNA level of miR-497/ NFkB1 /Ki67/ CD147/MMP-2 in tumors. **p < 0.01, compared to non-CSCs.

After confirming the biological function of CSCs and non-CSCs on cell proliferation *in vitro*, we explored the effect of CSCs and non-CSCs on tumor growth *in vivo*. As expected, the volume of xenograft tumor from CSCs cells were much larger than tumors from non-CSCs (Figure 10A, 10B). Moreover, the mRNA expression of miR-497/ NFkB1 /Ki67/ CD147/MMP-2 pathway in tumor were measured by RT-qPCR. Compared to the non-CSCs group, the mRNA expression of NFkB1 /Ki67/ CD147/MMP-2 were upregulated in CSCs group. But, miR-497 were decreased in CSCs group (Figure 10C). Those results suggest that CSCs promote tumor growth *in vivo* than in non-CSCs.

## DISCUSSION

Although a variety of therapies have been developed for treating cancer, they are of limited value in pancreatic cancer, and the overall 5-year survival rate of pancreatic cancer patients has changed little over the past several decades [38, 39]. As standard chemotherapy for pancreatic cancer, gemcitabine provides little benefit for most patients due to innate or acquired resistance. Various cytotoxic drugs and targeted agents have been employed in attempts to improve the sensitivity of pancreatic cancer cells to gemcitabine, but most had little value, leaving gemcitabine as the sole standard of care [39]. In the present study, we observed that miR-497 levels were downregulated in pancreatic cancer, especially in pancreatic CSCs. Furthermore, to the best of our knowledge, our current study is the first to reveal that the downregulation of miR-497 contributed to gemcitabine resistance and that overexpression of miR-497 significantly enhanced gemcitabine sensitivity in cancer cells, including CSCs and non-CSCs. In addition, we proved that miR-497 inhibition could also enhance the migration and invasion of pancreatic CSCs and non-CSCs.

Increasing numbers of studies have demonstrated that CSCs play a critical role in the initiation, maintenance, therapy resistance, and reoccurrence of cancer [40]. In the present study, our data showed that increased miR-497 expression was associated with a decrease in the sensitivity of pancreatic CSCs to gemcitabine. Moreover, this effect on gemcitabine resistance was also observed in pancreatic non-CSCs. These findings indicated that miR-497 inhibition might enhance gemcitabine sensitivity not only in CSCs but also in non-CSCs. The similarity of these effects might be due to CSCs and non-CSCs having the same mutational/genetic background as a result of their hierarchical organization [41]. The survival of CSCs depends on their microenvironment, which includes non-CSCs and cytokines [42]. During stimulation, the stemness of non-CSCs is promoted [43]. Therefore, a therapeutic method that targets both CSCs and non-CSCs might be better than a method that only targets CSCs.

Therapies based on miRNAs are viewed as promising methods for treating cancers. Consistent with the role played by miR-497 in gemcitabine sensitivity, it has been noted that miR-497 decreases cell proliferation and the percentage of S phase cells, resulting in an increase in sensitivity to gemcitabine, and also decreases in cell migration and invasion [24]. In addition, the promoting effect of miR-497 on cell sensitivity to chemotherapy has been observed in a variety of cancer cells, including neuroblastoma cells, colorectal cancer cells, and cervical cancer cells [44–46]. The primary mechanism underlying this sensitization effect is the ability of miR-497 to regulate signaling pathways associated with cell proliferation, apoptosis, and the cell cycle [44]. This is because many direct targets of miR-497 are cell cycle regulators (CCNE1, CDC25A, CCND3, CDK4), apoptosis-associated proteins (BCL2 and BCL-w), or proliferation factors (FGFR1/2, IGF-1R, and mTOR) [44, 47]. However, there are still limitations to this study. Most of the experiments in this study focus on cells, and there are few studies on clinical tissues; thus, our conclusions lack the support of clinical data. This study is a preliminary study on the role and mechanism of miR-497 in pancreatic cancer, which needs to be further explored.

As a master transcription factor, NF-κB is constitutively activated in many types of cancer and thus contributes to cancer development [33]. Increased NF-κB activity plays an important role in the acquisition of chemotherapy resistance in certain cancers, including pancreatic cancer [48]. Additionally, it was observed that translocation of NFκB1 into the nucleus is facilitated by Annexin A2 and subsequently leads to gemcitabine resistance in pancreatic cancer [33]. Consistent with these findings, we found that the levels of NFκB1 expression in pancreatic cancer CSCs were significantly increased, and ectopic expression of NFκB1 could induce gemcitabine resistance in CSCs and non-CSCs. Another recent study showed that NFκB1 was also positively associated with gastric carcinogenesis [49]. Our luciferase reporter assay showed that NFκB1 was a direct target of miR-497. Cells transfected with miR-497 mimics and subsequently treated with gemcitabine displayed decreased viability, metastasis, and invasion compared to nontransfected cells. However, viability, metastasis, and invasion were increased in mimic-transfected cells compared to NC vector-transfected cells. These findings indicated that the acquisition of gemcitabine resistance was suppressed rather than abolished by the miR-497 mimics. This might have resulted from an inability of the miR-497 mimics to suppress NFκB1 activity to the degree needed to abolish its biological function.

In summary, we report for the first time that the levels of miR-497 and NFκB1 expression were higher in pancreatic CSCs than in pancreatic non-CSCs. Our findings not only reveal the potential mechanism underlying the innate and acquired resistance to gemcitabine in pancreatic cancer cells, including CSCs and non-CSCs, but might also provide important information for developing methods that increase the sensitivity of pancreatic cancer to gemcitabine.
